# Glial scarring around intra-cortical MEA implants with flexible and free microwires inserted using biodegradable PLGA needles

**DOI:** 10.3389/fbioe.2024.1408088

**Published:** 2024-07-22

**Authors:** Fannie Darlot, Paul Villard, Lara Abdel Salam, Lionel Rousseau, Gaëlle Piret

**Affiliations:** ^1^ Braintech Laboratory, Institut National de la Santé et de la Recherche Médicale U1205, Université Grenoble Alpes, Grenoble, France; ^2^ ESIEE – ESYCOM Université Paris Est, Noisy-le-Grand, France

**Keywords:** biocompatibility, implants, neural recording, microelectrodes, flexible, microwires, glial scar, biodegradable

## Abstract

**Introduction:** Many invasive and noninvasive neurotechnologies are being developed to help treat neurological pathologies and disorders. Making a brain implant safe, stable, and efficient in the long run is one of the requirements to conform with neuroethics and overcome limitations for numerous promising neural treatments. A main limitation is low biocompatibility, characterized by the damage implants create in brain tissue and their low adhesion to it. This damage is partly linked to friction over time due to the mechanical mismatch between the soft brain tissue and the more rigid wires.

**Methods:** Here, we performed a short biocompatibility assessment of bio-inspired intra-cortical implants named “Neurosnooper” made of a microelectrode array consisting of a thin, flexible polymer–metal–polymer stack with microwires that mimic axons. Implants were assembled into poly-lactic-glycolic acid (PLGA) biodegradable needles for their intra-cortical implantation.

**Results and Discussion:** The study of glial scars around implants, at 7 days and 2 months post-implantation, revealed a good adhesion between the brain tissue and implant wires and a low glial scar thickness. The lowest corresponds to electrode wires with a section size of 8 μm × 10 μm, compared to implants with the 8 μm × 50 μm electrode wire section size, and a straight shape appears to be better than a zigzag. Therefore, in addition to flexibility, size and shape parameters are important when designing electrode wires for the next generation of clinical intra-cortical implants.

## 1 Introduction

Microelectrode arrays (MEAs) are important because of their high precision in recording the activity, or action potential, of an individual neuron, both *in vivo* and *in vitro* (Piret et al., 2015; Wu et al., 2021; Zeng and Huang, 2023). Efforts have been made to increase the efficacy and sensibility of electrodes or sensors in neural recording (Hébert et al., 2018; Viana et al., 2024) or to increase the number of recording sites to better understand the neural circuitry (Steinmetz et al., 2018; Schaefer et al., 2020). Concerning clinical applications and a better understanding of brain diseases, including neuro-ethical considerations (Bocquelet et al., 2016), one of the main challenges for MEAs penetrating neural interfaces (PNIs) is to limit the immune response they trigger in order to keep delivering qualitative neural signals including action potentials. Ideally, the same electrode remains connected to the same neurons over time (Barrese et al., 2013; Kozai et al., 2015; Woeppel et al., 2021; Zhao et al., 2023). Electrodes that connect to different neurons complicate the signal analysis and impair the reproducibility and use of records for the patient rehabilitation system (Cham et al., 2005; Ganguly and Carmena, 2009). The foreign body response post-implantation affects implant functioning in both the short and the long term (Polikov et al., 2005; Williams, 2008; Fernández et al., 2014; Prodanov and Jean, 2016; Carnicer-Lombarte et al., 2021). Persistent inflammation leads to neuronal death around the implant, thus causing signal loss and neurodegeneration (Zhang et al., 2021). The brain has a separate immune system from the rest of the body; it is protected by the blood–brain barrier (BBB) that keeps bacteria or infections from reaching the brain (Banks, 2015). Many brain cells intervene in injury healing (Scadden, 2006; Nishiyama et al., 2009). However, microglia and astrocytes are the most significant when implanting neural electrodes. They cause the main inflammatory responses in the brain, although this is not their only role.

Microglia, as the first responders to a foreign body, rapidly change their shape to an “amoeboid/hypertrophic morphology” (Savage, Carrier, and Tremblay, 2019). They proliferate and circulate to clean debris and encapsulate the device. Microglia interact chemically and mechanically with their environment; they are susceptible to both chemical signaling and mechanical signals, such as the stiffness gradients in the tissue surrounding neural implants (Bollmann et al., 2015). This is also one reason why rigid devices such as silicon-based neural implants induce a high immune response, which is a limitation for clinical applications requiring long-term neural recording (Barrese et al., 2013; Woeppel et al., 2021). Examples of such devices are the Utah array (Maynard et al., 1997), the Michigan probe (Kindlundh et al., 2004), and the NeuroNexus matrix arrays (NeuroNexus Tech. Co., 3D probes). One study has shown that the foreign body reaction around the implantable neuroprostheses leads to the accumulation of proteins in the same manner as the accumulation of pathological amyloid proteins for Alzheimer’s disease (Wellman et al., 2023), which makes biocompatibility a crucial aspect, even more so when an MEA is used as a diagnostic tool (Venkatesh et al., 2019). During normal conditions, astrocytes usually encapsulate blood vessels and contribute in synapses by recycling some neurotransmitters; they maintain good health for the neurons by providing energy and modulating their environment, ionic composition, and pH (Sofroniew and Vinters, 2010). Astrocytes respond to microglial signaling 1 week after implantation; they change their shape, encapsulate the device, and release factors that further promote the foreign body reaction (Vainchtein and Molofsky, 2020). Four to 6 weeks post-implantation, although they are involved in tissue healing and regeneration, they form a dense scar around the device, called the glial scar, that alters the proper neuronal integration on the electrodes and neural unit signal acquisition (Fawcett and Asher, 1999; Barrese et al., 2013).

The literature abounds with evidence that a successful brain implant must mimic the brain microenvironment for it to be efficient in the long term, notably in terms of material stiffness, shape, and surface chemistry. Carbon-based microfiber electrodes demonstrated improved signal-to-noise ratios because of their flexible interaction with the brain’s microenvironment (Hejazi et al., 2021; Faul et al., 2024). Matrices such as mesh-like implants can merge with the brain tissue and show a low immune response (Zhou et al., 2017). In addition, the interface at the membrane level of cells can be optimized with a nonplanar and, therefore, more biomimetic shape for the implant or the microelectrodes (Piret and Prinz, 2016; Mobini et al., 2022). It has been shown that gold microelectrodes with a “mushroom-like” structure improve neuronal integration and vascularization around the implant (Sharon et al., 2021), and 3D electrodes lead to better signal and spatial resolution (Wijdenes et al., 2021).

In the meantime, implant materials are susceptible to biodegradation and delamination over time, so that too-thin dimensions, under the micrometer range, remain questionable for a long-term strategy (Kozai et al., 2015). Here, we evaluated the biocompatibility of the three different electrodes that varied in dimension and shape for use as a brain implant. For clinical trials, more constraints and commodities in the sterilization and implantation strategies must be taken into account. We developed a method to implant our flexible and free MEA microwires using a needle made of poly-lactic-glycolic acid (PLGA). We have chosen PLGA as it biodegrades relatively slowly. The degradation rate of a material within brain tissue is a crucial point as a too-fast degradation rate could lead to a too-rapid accumulation of debris, inducing acute stress in microglia, leading to a chronic immune response, and a too-slow degradation rate could lead to microglia reactions over a too-long period, triggering chronic stress as well. PLGA biodegrades by hydrolytic attack *via* chain scissions of ester bond linkages, leading to lactic and glycolic acids, which are in turn eliminated through the blood–brain barrier and from the body as carbon dioxide and water (Alsaab et al., 2022). The material is FDA-approved for use in drug delivery purposes in the brain where the degradation rate of PLGA is suitable (Kou et al., 1997; Loureiro and Pereira, 2020; Alsaab et al., 2022).

## 2 Materials and methods

### 2.1 MEA implant fabrication and characterization

Hexamethyldisiloxane (HDMS) was spin-coated on a 4-inch silicon wafer previously sputtered with a TiAl (20 nm/200 nm) film (MEB550 PLASSYS equipment). SU8 2005 (CTS) was then spin-coated onto a silicon wafer for 50 s at 4000 rpm; baked at 65°C and at 95°C for 1 min and 2 min, respectively; exposed to UV light for 60 s (6 mW/cm^2^); and baked again at 65°C for 1 min and at 95°C for 2 min. SU-8 was then heated to 180°C for 5 min to cure the remaining solvent. Those SU8 substrates were sputtered with a Ti/Pt layer (50 nm/200 nm), and AZnlof 2070 was spin-coated (v3500, a2000, 50 s, annealed for 6 min at 100°C) and shaped (optical mask + M26 at 60 s) to define and protect the metal tracks, electrode, and connection pads during the IBE etching step. AZnlof was removed with acetone, and another layer of SU8 2005 was then spin-coated (50 s at 4000 rpm); baked at 65°C and at 95°C for 1 min and 2 min, respectively; and exposed with an optical mask to define the electrode and connection pads. SU8 2005 was then developed (60 s in SU8 developer, 15 s in IPA, water rinsed). A 120-nm layer of Al was deposited on this wafer, and AZnlof was processed to define an Al mask using an IBE etching step. The Al mask could define the final shape of the implant (microwire size; shape), and the SU8 2005 uncovered by the Al mask was removed using ICP etching (ICP-RIE Plasmalab100, OXFORD Instrument, UK). The Al mask was finally etched by IBE, and a scanning electron microscope (ZEISS ULTRA +SEM, Germany) was used at 2 KeV to perform electrode images with a tilt angle of 10°–20°. The thin, flexible MEA implants (SU8/Pt/SU8 stacking ∼ 8 µm thick) were detached from their wafer using a previously reported method of the aluminum layer electrochemical etching (Clément Hébert et al., 2016). For electrochemical potential measurement of the electrodes using cyclic voltammetry (CV), an aqueous solution of 1% phosphate-buffered saline (PBS, Sigma Aldrich) was used. The CV was performed using a Biologic SP200 potentiostat in a three-electrode setup where the studied microelectrode was the working electrode, an AgCl electrode was the reference, and a platinum mesh was the counter electrode. We compared the CV of our electrodes, which are made of Pt nanostructures, with the CVs of some conventional flat Pt microelectrodes having a similar electrode size. We also compared them with PEDOT:PSS microelectrodes, which are known for having one of the best CV results. Impedance measurements were performed at 1 kHz using a NanoZ (Multi Channel Systems GmbH, Reutlingen, Germany). Spike recording was performed using a Wi-Fi-system W2100 from MCS. The connecting part of the implant has the layout for insertion in a ZIF (FH43B-71-0.2SHW from Hirose Electric Co) but was thermo-pressed to an extension (polyimide/copper) to ensure several insertions in the ZIF component, the latest being part of a homemade PCB to send signals to two Omnetics (OMNETICS A79026-001), as required by the W2100 headstages.

### 2.2 MEA-PLGA needle fabrication

We linked the thin and flexible MEAs to PLGA needles for insertion. A wafer of silicon was patterned using Deep RIE to obtain the desired shapes of silicon needles (with a thickness of about 130 µm) in order to make a PDMS mold (Sylgard^®^ 184, Dow Corning, 1/10 ratio kit) with an inverted needle shape. This PLGA solution was made of a powder PLGA (Resomer^®^ RG 504 H, Poly (D,L-lactide-co-glycolide), Sigma 719900-5G), a polymer with a 50:50 ration of PLA: PGA, and the powder was dissolved with a ratio of 80 mg in 1 mL of anisole solvent. Note that the ratio is an important parameter as it can change the degradation rate, the tensile strength, and Young’s modulus of the copolymer, with more PLA leading to a slower degradation and a softer material (Jem and Tan, 2020). The molds were filled using drop deposition, and a razor blade was used to avoid a film forming outside the molds. The filled molds were placed in the oven for 40 min at 100°C and at room temperature for 2 h before unmolding. The final PLGA needles were obtained with a thickness of ∼10 µm, although they were ∼25 µm at the edge of the well, as shown in the image made with a scanning electron microscope (ZEISS ULTRA +SEM, Germany) at 2 KeV (Supplementary Figure S2). The PLGA needles were then stored in a dry environment. P4VP (Poly (4-vinylpyridine), Sigma)-coated glass slides were prepared (80 mg of P4VP in 2.5 mL of 70° ethanol spotting, annealing for 5 min at 50°C) to place previously autoclaved MEAs. Then, the PLGA needles on top using a binocular microscope. These mounted P4VP glass slides were annealed for 2.5 min at 100°C. Polylactides have a glass transition temperature (T_g_) and melting temperature (Tm) in the range of 50°C–65°C and 175°C–180°C, respectively, with higher L-lactide content contributing to increased transition temperature values. We deliberately used an annealing temperature above the Tg to attach the probes to the PLGA. The degradation of these PLGA needles was tested in a PBS solution at 37°C, and the needles were fully degraded after 35 days. Evaluating the tissue at 7 days and 2 months allowed determining that the degradation happens between those two time points. Figure 2 gives an indication of the PLGA volume at 7 days. The MEA-PLGA needles were then detached from the P4VP glass slide support in a 70% ethanol bath. Note that for clinical applications, PLGA needles could be prepared in sterile conditions under a sterile hood, such as for drug delivery, or alternatively, they could be sterilized with ethylene oxide gas, although that strategy would need to be checked for biocompatibility. Finally, to achieve its insertion by a motorized micromanipulator (Narishige, DMA-1511), each MEA-PLGA needle was placed on a sterile plastic support. The opposite side of the PLGA tip was stuck to this plastic support with a poly-ethylene-glycol (PEG) drop. After the MEA-PLGA insertion, the PEG drop is dissolved and rinsed long enough in a physiological saline solution to release it from the plastic support. The needle base is 900 µm wide, and the last 3 mm of PLGA needles is inserted to implant MEA intra-cortically (Supplementary Figure S2).

### 2.3 MEA implantation

Experiments were performed on young adult Sprague–Dawley rats (250–300 g, 2–3 months; Charles River Laboratories, France). The animals were handled and cared for in accordance with the European Communities Council Directive of 22 September 2010 (2010/63/EU, 74). Experimental protocols were approved by the Animal Ethics Committee COMETH (C2EA n°12, Grenoble) and by the French Ministry for Research (protocol number Apafis 04815.02). All efforts were made to ensure animal wellbeing and minimize animal suffering while optimizing data output. Animals were housed under controlled temperature (21°C) and light (12-h light/12-h dark cycle) conditions, with food and water provided *ad libitum*. For MEA implantation, rats were sedated with isoflurane (Vetflurane) and then anesthetized by an injection of ketamine/xylazine (Imalgene 1000/Rompun 2%, 100 mg/kg and 5 mg/kg, respectively; i.p.), and were treated with Rimadyl (4 mg/kg, carprophene 50 mg and benzyl alcohol 10 mg; s.c.) and NaCl 0.9% (2 mL; s.c.). The animals were then placed in a stereotaxic frame, their body temperature was controlled using a heating system (37°C ± 0.5°), and their eyes were protected with Lacrygel. The rat’s head was shaved and washed with betadine (Vetedine), and local anesthesia of the scalp was performed by injection of xylocaine (Xylovet, 0.1 mL; s.c.). The skin was incised, the cranial bone was cleaned of adhesions, and a rectangular craniotomy (6.4 mm × 3.7 mm ± 0.3 mm) was performed on the right side. The piece of bone was preserved during surgery in a physiological solution. A small incision was made in the dura mater, and the MEA-PLGA needle was inserted using a motorized micromanipulator (200 μm/s, 3 mm deep, Narishige, DMA-1511) with the stereotaxic coordinates of each MEA microwire type being referenced. The piece of bone was put back into place, bone wax was placed to seal the craniotomy area, and the skin was sutured and cleaned. For connection to the recording system, SU-8 insulated wires were protected by a silica gel (Kwik-Sil, WPI) and a homemade 3D printed cover with two parts (a fixed base and a removable cover). Animals received glucose (5%, 0.5 mL, s.c.) before they were housed singly for recovery. Clinical monitoring was carried out in order to preserve the wellbeing of the animals, and treatment of symptoms was carried out if necessary (Rimadyl 4 mg/kg, adapted food, water intake, and environmental enrichment).

### 2.4 Tissue processing and histology

**FIGURE 1:**
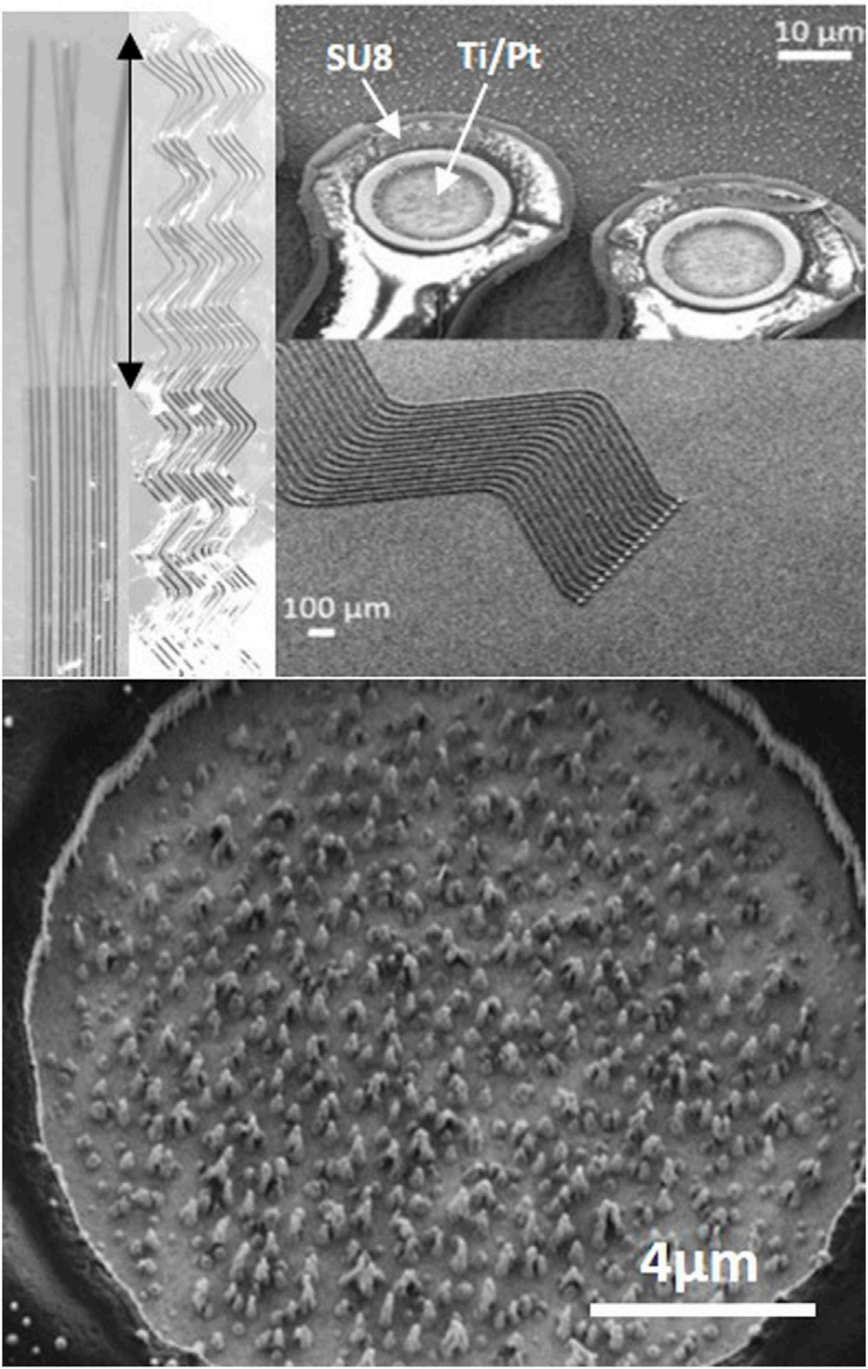
Neurosnooper MEA micro-wire implant. Top left: image at binocular showing the design with straight (S10) or zigzag (Z10) 10 μm large (and 8 μm thick) micro-wires and the 2 mm long implantable part for intra-cortical insertion (indicated with a double side arrow). Top right: SEM microscopy of the electrodes on the silicon wafer before being released and observed at binocular. Bottom: SEM microscopy of the Pt nanowire electrode (15 μm diameter).

We performed a time-dependent histology study of implant/brain tissue interface to evaluate the rat brain’s immune response to three different MEAs that varied in terms of the size and shape of the microwires at both 7 days and 2 months post-implantation. Two different implant shapes were studied, with S corresponding to a straight electrode shape and Z corresponding to a zigzag shape. In addition, the numbers next to the S or Z correspond to the respective electrode widths, 50 and 10 µm (Figure 1), whereas the thickness of the stacking was 8 µm for all electrodes. For each sacrifice time, three rats were implanted for each electrode width and shape. After the corresponding time lapse following MEA implantation (7 days or 2 months), the rats were deeply anesthetized (Dolethal, 150 mg/kg) and perfused transcardially with saline (for exsanguination), followed by formalin solution (stabilized PFA 4%). After perfusion, the brain was carefully dissected and stored in the same fixative for 48 h at 4°C. Post-fixation, the brain samples were stored in PBS at 4°C. Rat brain tissues were then sliced horizontally using the Mikrotom Mit VT1200/VT1200S (Leica Biosystems) at a speed of 0.22 mm/s to obtain 50-µm-thick brain sections. Sections were collected as quickly as possible to avoid losing microwire implants in the PBS bath. However, after microtome brain cutting, not all wire fragments were found where they could be moved out of the brain slice. Indeed, to perform a brain section with the Mikrotom, the brain is placed in a Bath with ∼50 mL of PBS, and after cutting, although we collected the sections as fast as possible, it is possible that some wires detached and were lost in the cuve liquid. We imaged and labeled all sections, including those where the wires were found and the others. The sections were then stained with primary antibodies for GFAP and IBA1, respectively; goat antibody from Abcam (ab 53,554; diluted 1:1000) and rabbit antibody from FUJIFILM Wako Pure Chemicals Corp. (019–19741; diluted 1:200) with an incubation time of 3 days at 4°C. Their respective secondary antibodies were donkey anti-goat antibody from Abcam (ab 150,133, diluted 1:1000) and donkey anti-rabbit antibody from Life Technologies Co., and sections were incubated with these in the dark for 2 days at 4°C. Finally, DAPI staining solution (ab 228,549, diluted 1:1000) from Abcam was used to stain cell nuclei, and the sections were mounted between a glass slide and a coverslip.

### 2.5 Image acquisition and quantification

For quantitative analysis, fluorescent images of the stained 50-µm brain sections were captured by the upright Olympus BX51WI Fixed-Stage microscope at the BrainTech lab. The 1920 × 1080 pixel image files were in *.tif format in the RGB color space. Calibrations in x and y were both 578.029 nm/pixel with a 0.3 numerical aperture. CellSens standard software was used to acquire the images and set the same exposure time for each antibody. The bright field light was used to localize the MEA microwire implant and center it, and then three excitation wavelengths, UV, blue, and green, were used sequentially for the imaging of DAPI, GFAP, and IBA1 labeling emitting blue, green, and red fluorescence, respectively. For qualitative analysis, some chosen samples were imaged using the Zeiss LSM 710 confocal microscope at the Grenoble Institute des Neurosciences (GIN). The inverted microscope had a 0.5 numerical aperture, a resolution of 250 nm in x and y, and a resolution of 500 nm in z. The Z-stack acquisition mode was used. The 1024 × 1024 pixel images were captured in *.czi format. The average intensity of markers for each condition (days post-implantation, implant type) was found for several areas (82.08 µm × 519.08 µm) chosen from the center of scars, with and without wires, that were found in the slices from three rat brains. The quantification of the chosen images was done using ImageJ, which is a Java-based image processing software, and the plot profile tool was reported in an Excel Spreadsheet. The average intensity and standard deviation of all images were calculated and plotted. A *t*-test was calculated, using Excel’s embedded function “ = T.test”, for the statistical significance of the compared data, which led to a near-zero value. The measurements of the glial scar cavity were made by assuming an elliptic shape for the scar and measuring its major and minor radius from the glial scar center.

## 3 Results

### 3.1 Biodegradable needle for intra-cortical insertion of MEA thin flexible microwire

The Neurosnooper MEA implants were produced in a clean room and characterized before and after detaching them from the silicon wafers using a scanning electron microscope and a binocular microscope, respectively (Figure 1). Two different MEA implant shapes were studied with S corresponding to a straight electrode and Z corresponding to a zigzag shape. The numbers 50 or 10 in the electrode names correspond to the respective electrode widths, 50 µm and 10 μm, whereas the thickness of the stacking was ∼8 µm for all electrodes. Pt nanowire structures could be observed inside the 15-µm diameter electrodes, which were formed during the very last step of the IBE etching for Al mask removal. Impedances of these Pt nanostructured electrodes were 1.8 MOhm ±0.1 at 1 KHz. Cyclic voltammetry was performed, showing a charge storage capacity for the Pt nanostructured electrodes that was higher than classical Pt MEAs but lower than MEAs electroplated with PEDOT:PSS. After MEA characterization and sterilization, our thin flexible MEA microwires were assembled to PLGA needles in order to perform their intra-cortical insertion and allow for action potential recording (Supplementary Figure S1). PLGA is an FDA-approved polymer that biodegrades at a desirable rate to be used for drug delivery. It dissolves in the brain's aqueous environment (Makadia and Siegel, 2011). We are able to design PLGA needles with any desired shape using microlithography and etching steps, and MEAs were attached to the PLGA needles. Supplementary Figure S2 shows their successful insertion in agarose 0.6%, a mechanical brain tissue-like material (Chen et al., 2004). This insertion can be realized either manually or using a motorized micromanipulator from Narishige. We attempted to remove the PLGA needles 30 s after their implantation to check their stiffness and whether they could be implanted again; it was not possible as the PLGA needles had already softened. For the study follow-up, we used the motorized approach and performed a time-dependent histology study of the MEA implant and brain tissue interface to evaluate the rat brain immune response to PLGA needles without MEA and to PLGA transporting one of the three different types of MEA, at both short term (7 days) and long term (2 months) post-implantation.

### 3.2 PLGA needle degradation and tissue response

**FIGURE 2:**
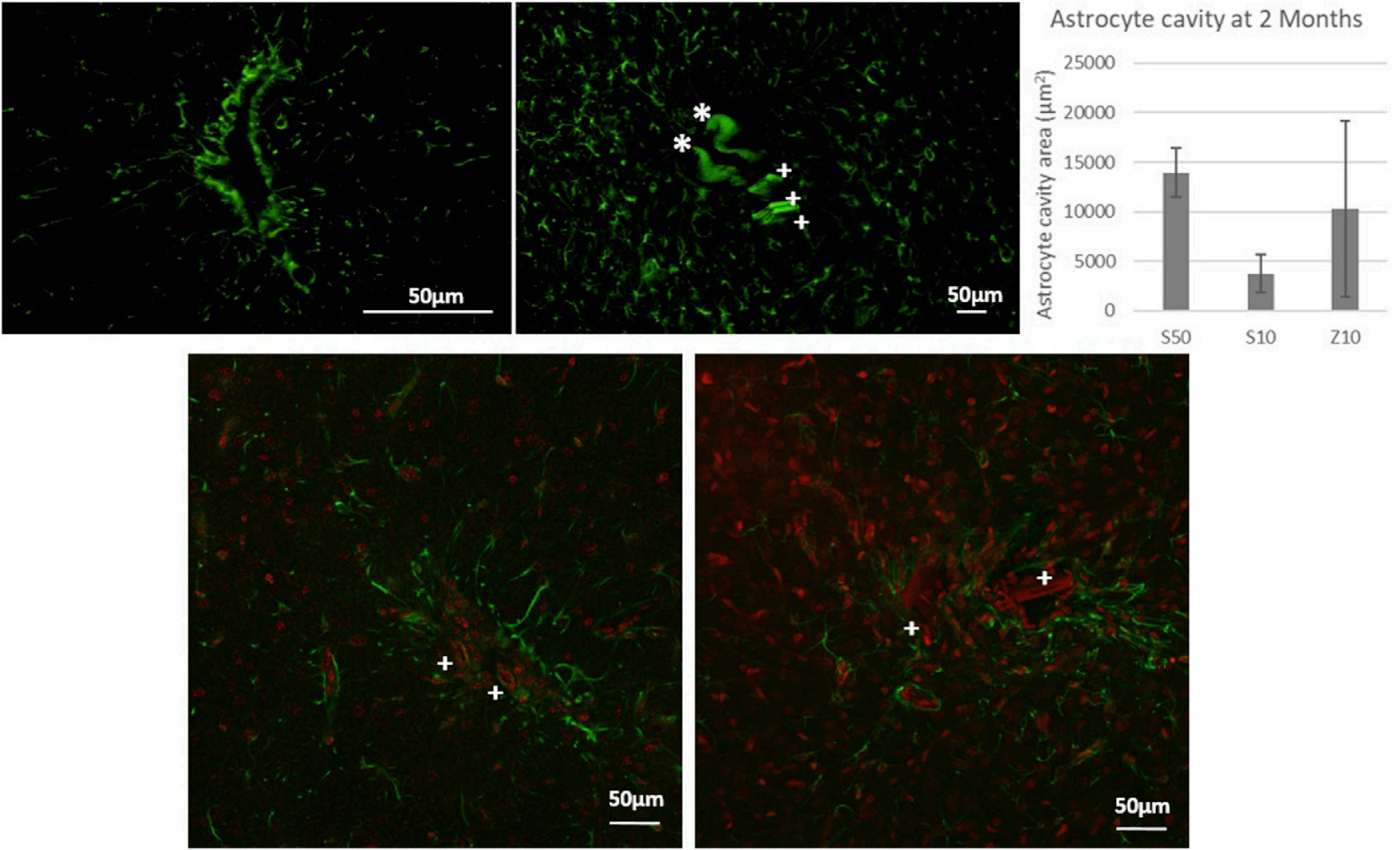
Fluorescence microscopy picture of GFAP labeling for control surgeries of the PLGA needle alone after 2 months of implantation (top left) and after 7 days with a MEA (top left: over-exposition to show the auto fluorescence in green of PLGA needle yet undissolved parts with a *, and of parts of 3 microwires found for the S50 MEA with a cross +). Glial scar cavities have been measured at 2 month post-implantation for the 3 MEA microwire types and standard deviation is represented as error bars. Overlay of fluorescence confocal microscopy pictures showing GFAP (green) and IBA1 (red) labelling around MEA microwire S10 (left) and MEA microwire S50 (right) at 2M post-implantation, and showing parts of MEA microwires (auto fluorescence in red) found with a cross +. Scale bar for all images = 50 μm.

We first performed control surgeries to evaluate the brain immune tissue response to the PLGA needle alone and its biodegradation effect after 2 months. These control surgeries showed brain tissue slices with a glial scar cavity that is less than 50 µm in all dimensions. Figure 2 shows an example of such a glial scar cavity, and all scars tend to have an elliptical or spherical shape, most probably due to the PLGA needle size of about 10 µm in one dimension and from 900 µm to a few µm in the other dimension. At 7 days post-implantation, we observe still-undissolved parts of PLGA needles that have an auto-fluorescence (Figure 2). The average intensities of the GFAP and IBA fluorescence imaging obtained from brain slices of rats that were implanted with the different MEA microwire sizes are reported in graphs. At this stage, the fluorescence intensity of GFAP labeling is slightly greater than the fluorescence background that can be observed far from the implantation, whereas the activity of microglia seems quite important (Supplementary Figure S3).

### 3.3 MEA microwires and brain tissue interface at 2 months post-implantation

#### 3.3.1 The effect of wire size post-implantation

**FIGURE 3:**
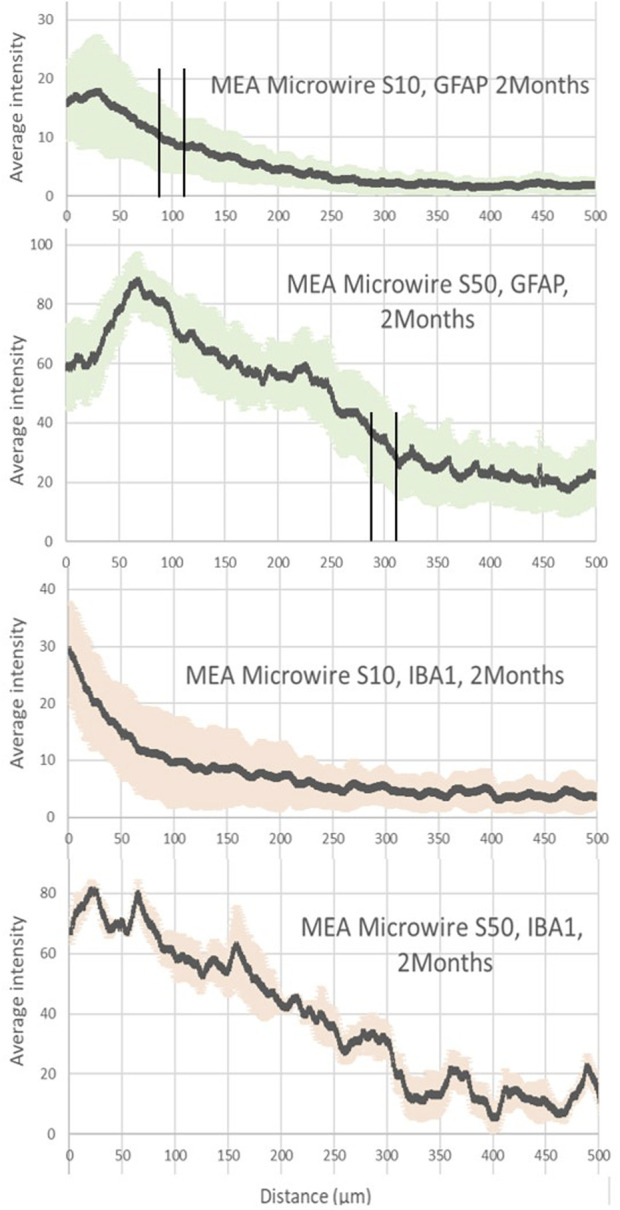
Average intensity of fluorescence microscopy of GFAP and IBA1, at 2 months post-implantation, showing respectively the astrocyte and the microglia distribution for the distance from the site of the different sizes of MEA microwire implants: straight MEA microwires with a section of 8 × 10 μm (MEA microwire S10) and straight microwires with a section 8 × 50 μm (MEA microwire S50). Standard deviation for each data point is represented as error bars in light green (GFAP) and light red (IBA1). We have spotted the thresholds for glial scar with an interval of ± 25 μm.

At 2 months post-implantation, confocal pictures show that qualitatively, astrocytes are more active and numerous around the 50-µm implants than around the 10-µm ones (Figure 2). In addition, quantitatively, in the first 100 µm near the MEA microwire, the maximum average fluorescence intensity of GFAP labeling in the brain slices of rats implanted with the MEA microwire S10 was found to be four times lower than that of the S50 (Figure 3). The average glial scar for the S50 size ends approximately 300 µm from the implant; for the S10 size, the scar ends 100 µm from the implant. Similar to the average intensity of GFAP, the maximum average intensity of IBA1 for the S50 MEA microwire is much higher than that of the S10 (Figure 3), and the microglia density decreases until approximately 100 µm from the S10 implant but only around 300 µm from the S50. In addition, the microglia activity decreased from day 7 to day 60 only for the S10 MEA microwire (Figure 3; Supplementary Figure S3). Both qualitative and quantitative studies validated that the 10-µm SU-8 electrodes elicited less immune cell activation than the 50-µm electrodes, although they have the same thickness of about 8 µm. The areas of the glial scar cavity could be measured by assuming an elliptical shape for the glial scars in all samples, and the areas are reported in Figure 2. This shows that thicker glial scars are associated with a larger glial scar cavity and confirmed that the S10 MEA microwire implants lead to smaller footprints of 3730 ± 1887 μm^2^ in the long term than the S50 MEA microwires, which have footprints of 13,927 ± 2471 μm^2^.

#### 3.3.2 The effect of wire shape post-implantation

**FIGURE 4:**
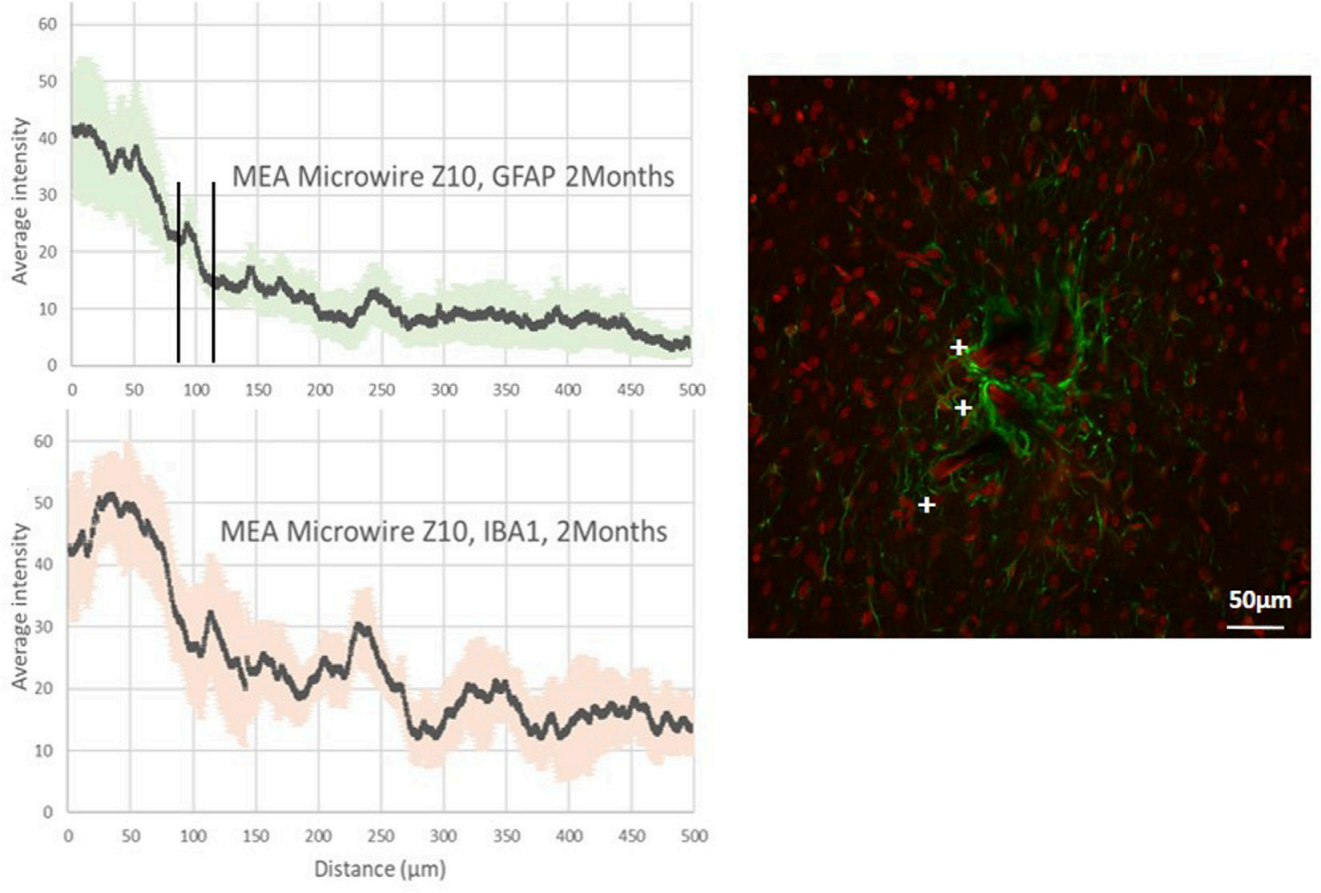
Average intensity of fluorescence microscopy of GFAP and IBA1, at 2 months post-implantation, showing respectively the astrocyte and the microglia distribution for the distance from the site of implants with ZigZag MEA microwires of a section of 8 × 10 μm (MEA microwire Z10). Standard deviation for each data point is represented as error bars in light green (GFAP) and light red (IBA1). We have spotted the threshold for glial scar with an interval of ± 25 μm. Overlay of fluorescence confocal microscopy pictures showing GFAP (green) and IBA1 (red) labelling around MEA microwire Z10 at 2M post-implantation, and showing parts of MEA microwires (auto fluorescence in red) found with a cross +. Scale bar = 50 μm.

Figure 4 reveals a maximum average fluorescence intensity of GFAP labeling two times greater for the Z10 MEA microwire with the zigzag shape than for those with the same width but a straight shape, the S10 MEA microwire, as displayed in Figure 3. The glial scar thickness is found, however, to be about 100 µm for both. Fluorescence intensity graphs of IBA1 tend to show that the Z10 MEA microwires trigger more microglial activity than the S10 MEA microwires but less than the S50 MEA microwires. Finally, although with an important error deviation bar, the average glial scar cavity measurement for the Z10 MEA microwires follows the same trend. It is 10,269 ± 8,849 μm^2^, which is larger than the glial scar cavity of the S10 MEA but smaller than the cavities of the S50 MEA microwire implants.

## 4 Discussion

### 4.1 The PLGA biodegradable needle as a surgical strategy for thin flexible MEA microwire insertion

Lately, efforts have been made to use MEA with flexible materials to reduce the stress due to mechanical mismatch and displacement at the neuron–electrode interface. This implies surgical methods to allow flexible material insertion, which can itself induce a chronic inflammation depending on the cell and blood vessel damage during the short time of surgery. To these engineering constraints of obtaining a small glial scar in the long term, which is an important first step to achieve long-term action potential recording, other constraints can be added for clinical applications, such as the compatibility to sterilization or the convenience for use by clinical surgeons. Our strategy using the coupling of our Neurosnooper MEA microwire (Figure 1) to PLGA needles aimed at integrating these requirements as efficiently as possible. Our results show small glial scar thicknesses and cavities in control surgeries 2 months after the PLGA needle insertion with a small footprint left of less than 50 µm (Figure 2), although the initial PLGA needle size is approximately 10 µm in one dimension and from 900 µm to a few µm in the other dimension. Several techniques were used to insert tiny flexible PNIs (Gu et al., 2021; Wang et al., 2023). Although a further study could show the neurons around the electrodes as well as their spiking activity, our results for the glial scar do not show a swelling as reported when the biodegradable material is carboxymethylcellulose (CMC) (Kozai et al., 2014). In the literature, 90 µm × 200 µm PVA/PLGA needles or 200 µm × 200 µm PLGA dissolving needles have already been reported by Pas et al. and Ceyssens et al., respectively, as a means to insert tiny probes for, respectively, acute and chronic neural recording (Pas et al., 2018; Ceyssens et al., 2019). We developed a method that is compatible with robotic insertion (a motorized micromanipulator from Narishige). It is also the first time that an MEA containing 60 microelectrodes is assembled with several PLGA needles, each containing 10 microelectrodes, which involved a slightly different method for PLGA-MEA assembling. In addition, this new MEA design allows for independent implantation locations with stereotaxic coordinates for each needle. Using such a biodegradable support rather than PEG (Lecomte et al., 2015; Gu et al., 2021) has at least two surgical advantages. First, only one travel is required as no second travel is needed to take the shuttle out, which is thought to be less invasive, and the fact that they become relatively soft within 30 s after implantation might avoid friction and mechanical stress to the tissue. Second, the slow degradation of PLGA makes it suitable for better precision to position sensors, even wireless sensors, within the cortex (McGlynn et al., 2021).

### 4.2 The role of the structure dimension and shape in flexible MEA microwire PNIs

This biocompatibility study aimed to evaluate the effect of different widths and shapes of the electrodes on the immune response. The S10 MEA microwires showed better biocompatibility than the S50 MEA microwires or the Z10 microwires (Figures 2–4). At 7 days post-implantation, a slight accumulation of astrocytes and an overexpression of microglia was observed for the 10-µm electrodes (Supplementary Figure S3). Two months after implantation, this overexpression is reduced, whereas the GFAP expression is slightly lower (Figure 3). This is compatible with the fact that astrocytes form a compact glial scar around the foreign body in the long term, whereas the microglia’s lamellipodia ensheathment happens at less than 2 months post-implantation (Salatino et al., 2017). In addition, the fact that reducing the size of the MEA microwire allows for smaller footprints in the tissue is in line with the volume effect described by Kozai et al. (2015) and with observations when using stiff implants (Seymour and Kipke, 2007; Seymour et al., 2011; Karumbaiah et al., 2013). To our knowledge, this effect has not yet been reported for soft implants, but our results align with those reported by Ceyssens et al. (2019), who used MEA microwires with a size of 1 µm × 300 µm and reported a similar glial scar thickness of ∼100 µm and very small footprints. Our results also show that the zigzag electrodes, although having the same width as the straight electrodes, displayed a similar glial scar thickness but higher fluorescence intensity of GFAP and IBA1 (Figures 3, 4), as well as a larger glial scar cavity (Figure 2). The idea of testing sinusoidal-shaped electrodes was based on the hope that they would compensate better for the micromovements of the brain and reduce the immune response (Sohal et al., 2014). The brain motion in rats is only about 10–30 µm during respiration and 10–60 µm during head movements (Gilletti et al., 2006), so we think that those results might be different in a study with larger animals.

## 5 Conclusion

In conclusion, many methods are being developed to further decrease the inflammatory response, such as bioactive coatings, surface nanostructuration, and the implant structure’s shape, dimensions, and materials. However, there is a compromise between the latter and the long-term stability of the materials, a mandatory perspective for long-term clinical applications. After a relatively short time, bioactive coatings could be removed, and nanostructures or too-thin structures could be biodegraded. Our results confirm that using structures with flexible materials and reducing their thickness and width seems critical. The ideal engineering process might be to obtain a thin, flexible structure that would resist erosion in tissues over time. Finally, we demonstrated a new approach in surgical techniques to insert such structures that would be compatible with ultra-thin or wireless structures and with sterilization processes required for clinical applications.

## Data Availability

The original contributions presented in the study are included in the article/Supplementary Material; further inquiries can be directed to the corresponding authors.
