# Supplementary figures and images for: Glial scarring around intra-cortical MEA implants with flexible and free microwires inserted using biodegradable PLGA needles
Source: Front Bioeng Biotechnol. 2024 Jul 22;12:1408088. doi: 10.3389/fbioe.2024.1408088 (PMC11298340; doi:10.3389/fbioe.2024.1408088)

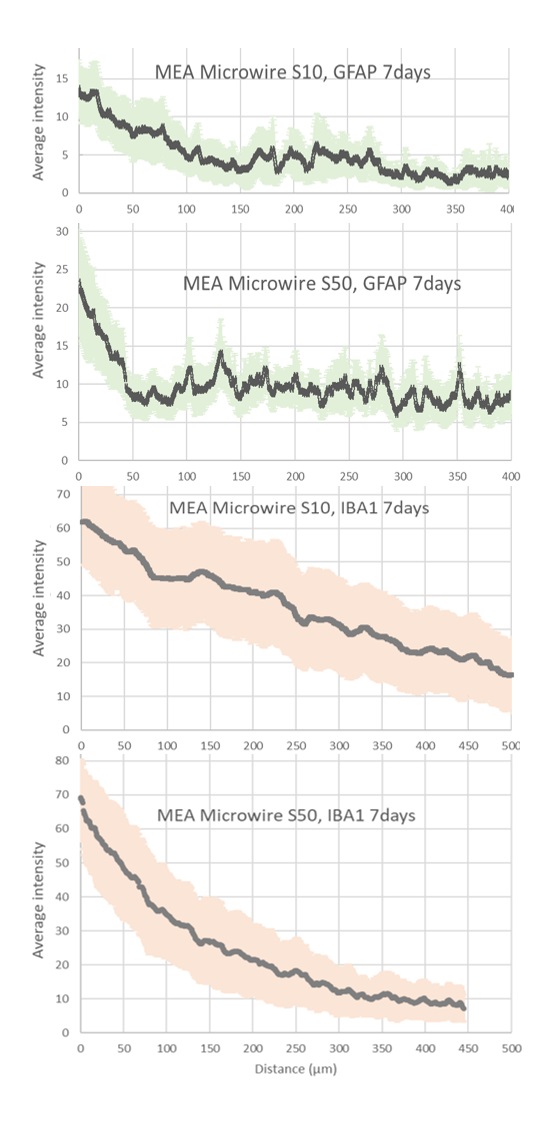

Supplement: Supplementary file 1 [file Image3.JPEG]

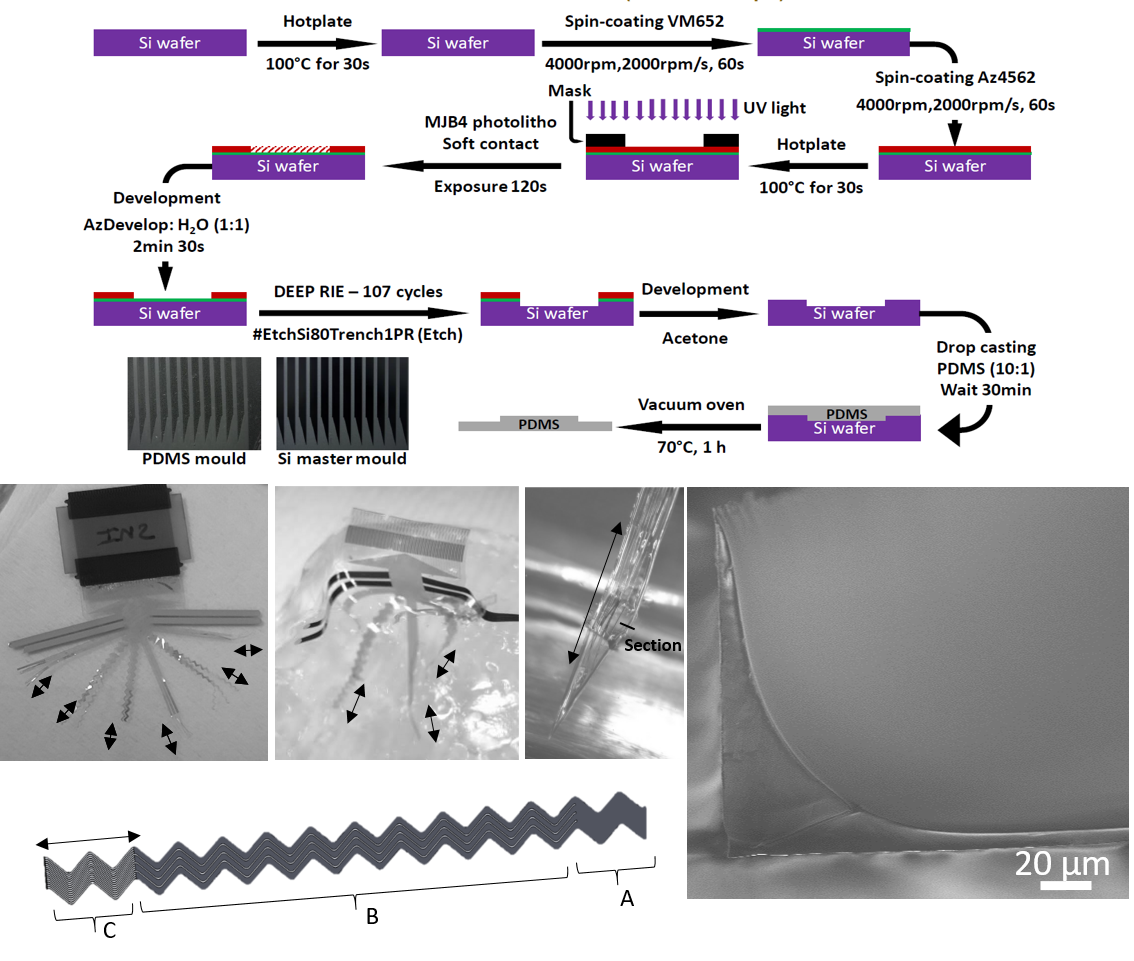

Supplement: Supplementary file 3 [file Image2.PNG]

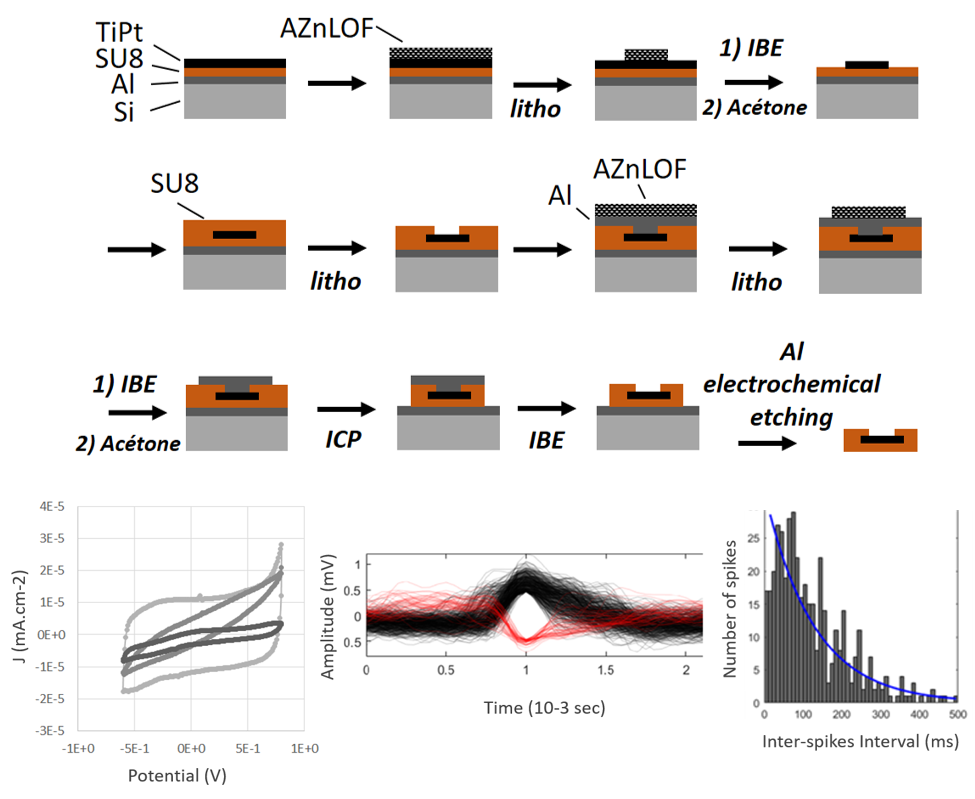

Supplement: Supplementary file 4 [file Image1.PNG]
