# Supplementary material for: Glial scarring around intra-cortical MEA implants with flexible and free microwires inserted using biodegradable PLGA needles
Source: Front Bioeng Biotechnol. 2024 Jul 22;12:1408088. doi: 10.3389/fbioe.2024.1408088 (PMC11298340; doi:10.3389/fbioe.2024.1408088)
Supplement: Supplementary file 2 [file DataSheet1.pdf]

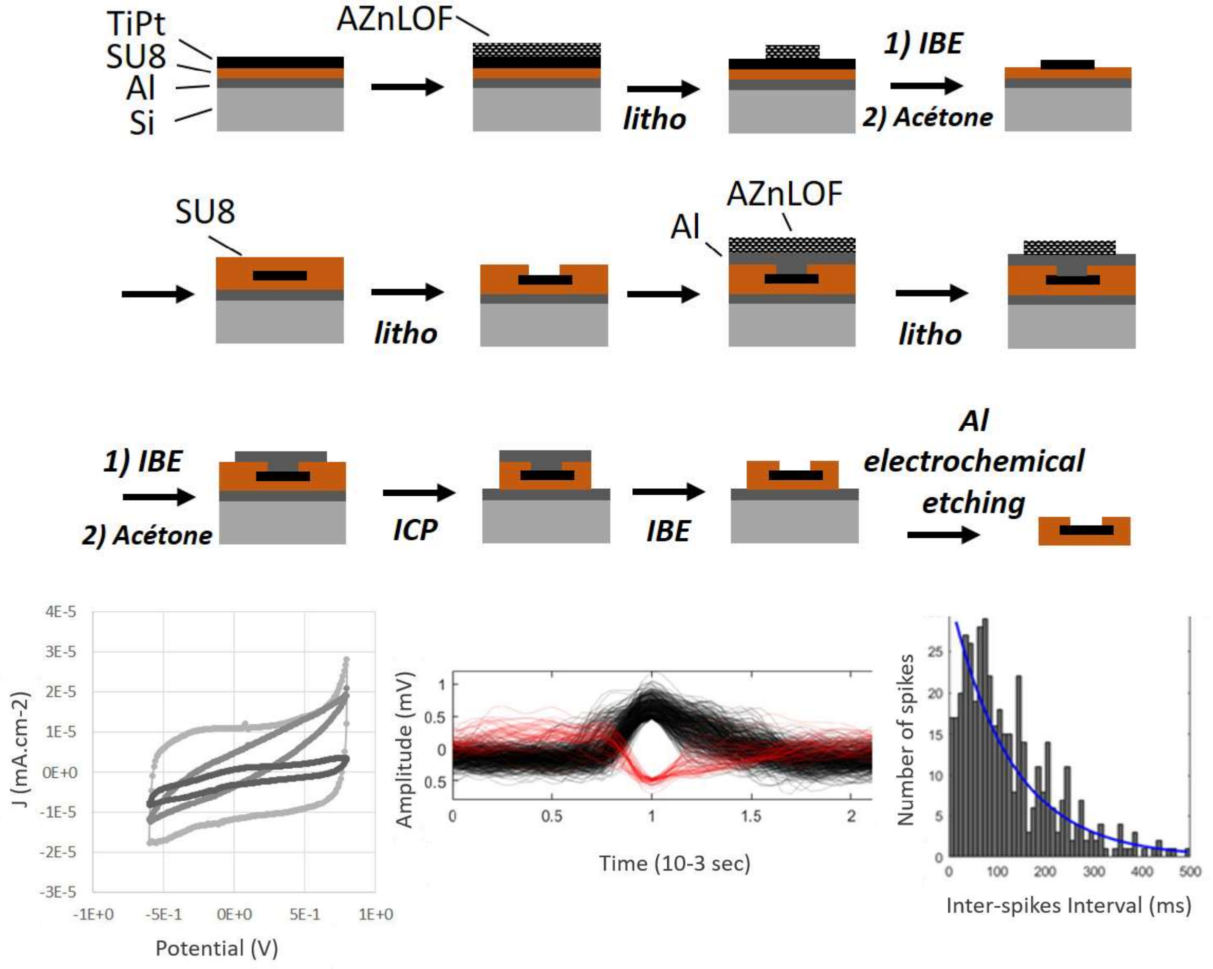

Supplementary Figure 1. Scheme of the implant fabrication steps. Cyclic voltammetry at 100 mV.s-1 in PBS for Neurosnooper MEA with nanostructured Pt electrodes of a 15µm diameter (grey), for MEA Qwane bioscience with Pt electrodes of a 20µm diameter (grey dark) and for MEA Qwane bioscience with PEDOT:PSS electrodes of a 15µm diameter (grey light). Example of two cell spiking activity and of inter-spikes interval that were recorded using the Neurosnooper MEA from day 15 after implantation.

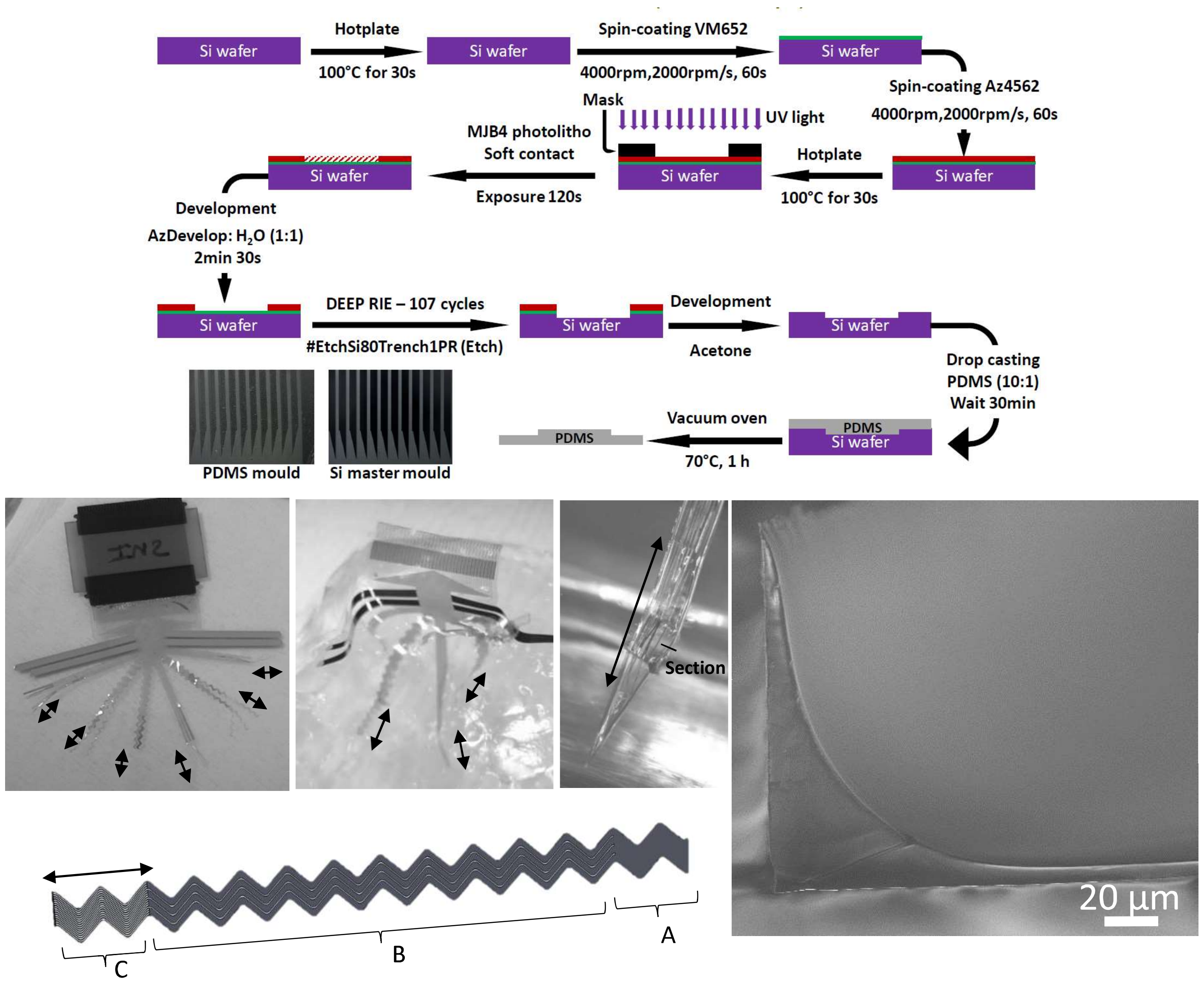

Supplementary Figure 2. Scheme for the PLGA needle fabrication steps. Picture of an implant connected to the extension adapted to a ZIF component (top left) and of an implant inserted in agarose 4% (top middle). Binocular microscopy picture of a PLGA needle assembled to a MEA during its insertion in agarose (top right). A scheme (bottom) showing the concept of the separation in gradually thinner wires in 3 steps : A- One wire with 10 electrode metal tracks; B- 4 wires of 2 to 3 electrode metal; 3- free between them MEA micro-wires. This separation allows a better mechanical resistance of the thin micro-wires during the implant manipulation (in liquid or air). The 2mm long implantable parts for intra-cortical insertion are indicated with double side arrows. A SEM picture of the PLGA needle cross section (section as indicated in the top right figure).

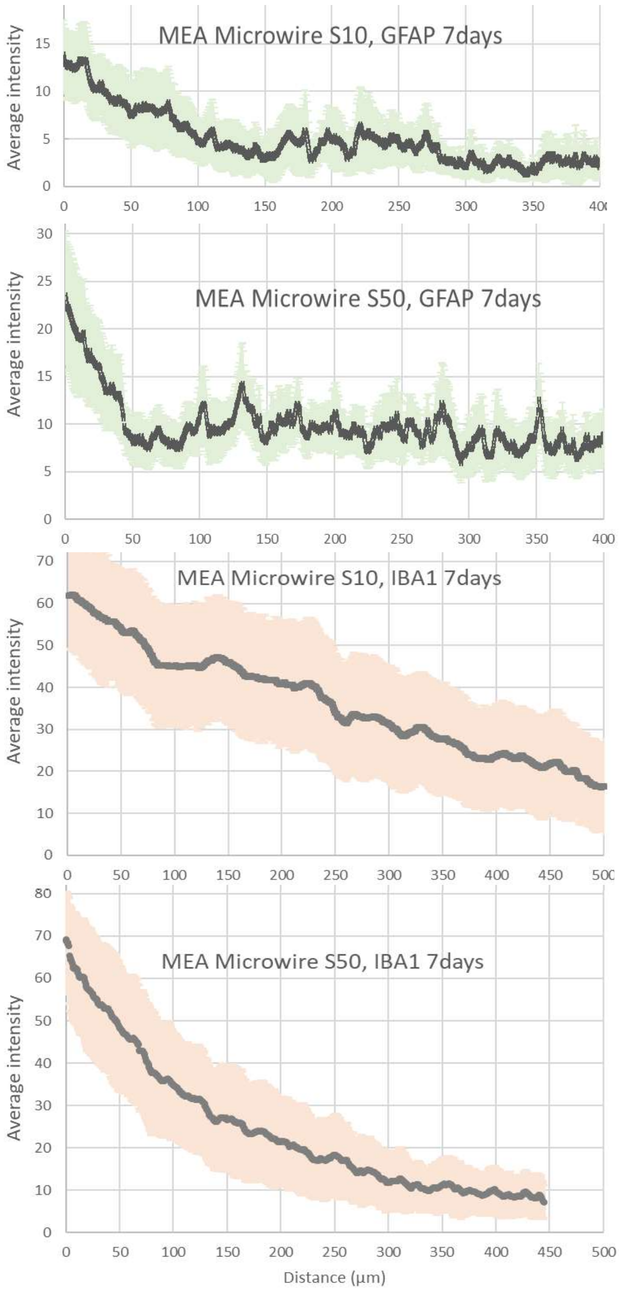

Supplementary Figure 3. Average intensity of fluorescence microscopy of GFAP and IBA1, at 7 days post-implantation, showing respectively the astrocyte and the microglia distribution for the distance from the site of the different sizes of MEA microwire implants: straight MEA microwires with a section of 8x10µm (MEA microwire S10) and straight microwires with a section 8X50µm (MEA microwire S50). Standard deviation for each data point is represented as error bars in light green (GFAP) and light red (IBA1).
